# Pediatric Meningeal Tumors of the Sylvian Fissure Region without Dural Attachment: A Series of Three Patients and Review of the Literature

**DOI:** 10.1055/s-0036-1584166

**Published:** 2016-05-26

**Authors:** Daniel Joseph Donovan, Varoon Thavapalan

**Affiliations:** 1 Department of Surgery, University of Hawaii–John A. Burns School of Medicine, Honolulu, Hawaii; 2 Department of Neurological Surgery, Thomas Jefferson University Hospitals, Philadelphia, Pennsylvania

**Keywords:** pediatric tumors, meningeal tumors, sylvian fissure meningioma, seizures

## Abstract

Pediatric meningeal tumors are rare, but those in the region of the sylvian fissure without dural attachment are extremely rare, with only 24 previously reported cases in the world literature. In this series, we report two additional cases of sylvian fissure meningioma without dural attachment and one case of perisylvian meningioangiomatosis in the medial temporal lobe. All three patients presented with complex partial seizures, but the diagnosis was delayed in each case because the symptoms were misinterpreted to be behavioral rather than epileptic. The seizures were eventually confirmed with electroencephalogram, and subsequent imaging showed enhancing masses within the sylvian fissure region that were at least partially calcified in all three cases. Each patient underwent craniotomy. In the first case, gross total resection was achieved, and in the second case, a small residual portion of tumor was densely calcified and adherent to the middle cerebral artery branches. Both of these were World Health Organization (WHO) grade I meningiomas. The third patient underwent biopsy and limited resection of meningioangiomatosis. No dural attachments were noted in any of the tumors, but one of the meningiomas was intraparenchymal in location, surrounding the sylvian fissure in both the frontal and temporal lobes, which has been described in only a small number of these cases previously. The patients underwent pre- and postsurgical neuropsychiatric testing and did not experience any significant cognitive deficits. At 10-year follow-up, the patient who had gross total resection of the tumor has had no recurrence and is seizure-free without anticonvulsant medications. The incompletely resected intraparenchymal meningioma in the second patient recurred after 5 years, however, and at repeat surgery was found to have transformed to a WHO grade II tumor. Radiation therapy was delivered and the tumor has been stable for 2 years, but the patient continues to have occasional seizures despite medication. The patient with meningioangiomatosis has had no further growth and has excellent control of seizures but remains on medication. We review the clinical presentation of these rare tumors and discuss the treatment, outcomes, and possible relationship of meningiomas to meningioangiomatosis.

Meningiomas are very common intracranial tumors in adults but are relatively rare in pediatric patients, comprising only 0.4 to 4.6% of all pediatric brain tumors in several large series. 1 2 3 4 Sylvian fissure meningiomas are extremely rare, with only 24 previously reported cases in the world literature, almost all occurring in children. 5 6 7 8 9 10 11 12 13 14 15 16 17 18 19 20 21 22 23 We report two additional cases of children with deep sylvian fissure meningiomas without dural attachment and an additional case of meningioangiomatosis (MA) in the perisylvian region. The literature is also reviewed regarding similarities and differences with the other small number of cases previously described.

## Materials and Methods

All three case histories and this manuscript were reviewed by our Institutional Review Board, which deemed the text acceptable for publication without a formal review. The case histories, images, operative reports, and follow-up notes were reviewed retrospectively.

## Case Reports

### Patient One

**Fig. 1:**
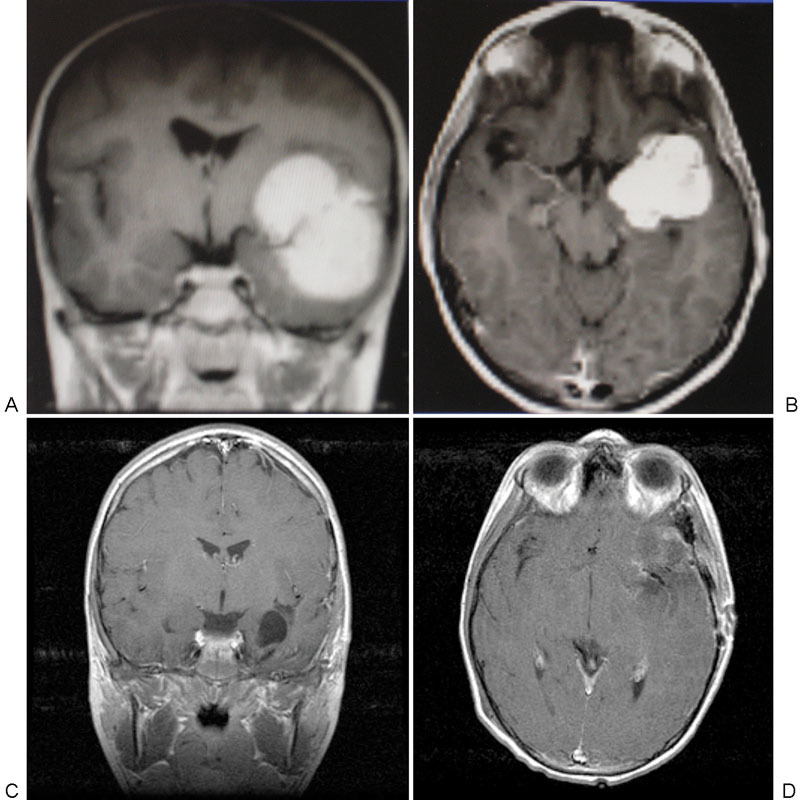
(A) Coronal and (B) axial contrast-enhanced preoperative magnetic resonance imaging (MRI) for patient one showing uniformly enhancing mass measuring 5 × 5 × 4 cm within the left sylvian fissure region. (C) Coronal and (D) axial contrast-enhanced postoperative MRI for patient one showing postoperative changes after microdissection and successful resection of tumor with no significant residual enhancement along resection margins. There has been no delayed recurrence of tumor in this patient at 10 years' follow-up.

An 11-year-old boy was referred for new-onset partial complex seizures occurring with increasing frequency over the previous 9 months. His mother described mild nausea, staring spells, and vague olfactory sensations during these episodes, which typically lasted 1 to 2 minutes and then resolved spontaneously. He was initially misdiagnosed with attention deficit disorder, because they occurred during school classes and caused him to lose concentration, but when treatment with methylphenidate was unsuccessful, he was also noted to have automatisms of his hands and lip-smacking, which eventually led to electroencephalogram (EEG) examination that revealed epileptiform activity in the left temporal lobe. Magnetic resonance imaging (MRI) then showed a 5 × 5 × 4-cm enhancing mass within the left sylvian fissure region ( Figs. 1A and 1B ), and he was then referred for surgical evaluation. Small areas of calcification were noted on computed tomography (CT).

He underwent a left frontotemporal craniotomy, and a grayish, relatively soft mass was found within the sylvian fissure, without any attachment to the dura. There was an easily discernible arachnoid plane surrounding the tumor, which was only mildly adherent in some areas to the middle cerebral artery (MCA) branches, where it was removed with careful microdissection. Postoperative imaging showed no residual tumor ( Figs. 1C and 1D ). The pathologic examination revealed a biphasic pattern of meningothelial cells and fibrous elements with negligible mitotic activity, consistent with World Health Organization (WHO) grade I meningioma. The patient remained asymptomatic and was weaned off anticonvulsant medications. Follow-up neuropsychiatric testing revealed no significant changes in cognitive functioning. The tumor has not recurred in over 10 years of follow-up imaging.

### Patient Two

**Fig. 2:**
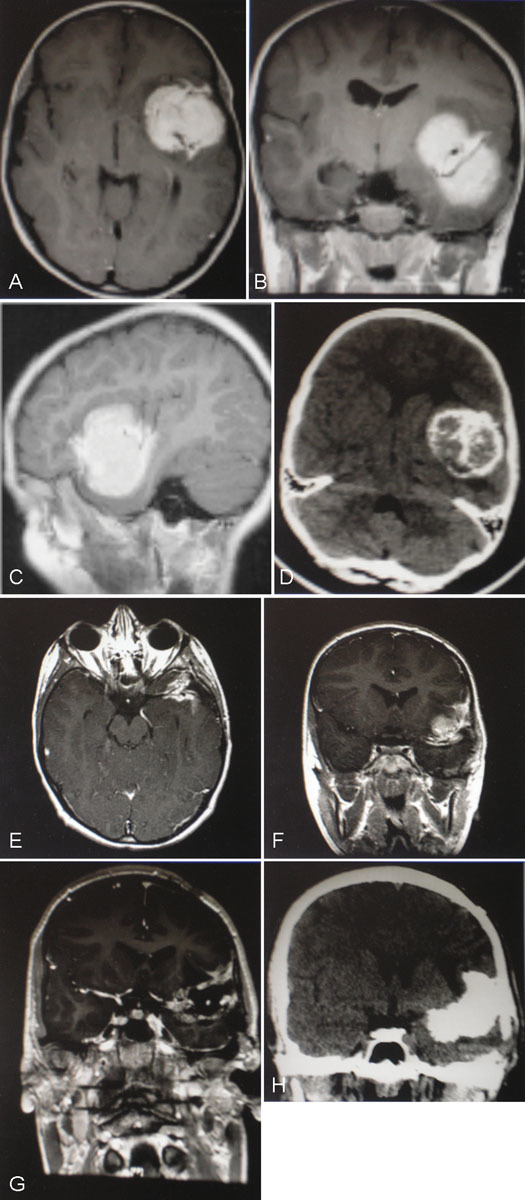
(A) Axial, (B) coronal, and (C) sagittal contrast-enhanced preoperative magnetic resonance imaging (MRI) for patient two. A 4 × 5 × 4-cm homogeneously enhancing mass is present within the left sylvian fissure, which is densely calcified on noncontrast computed tomography (CT) (D). (E) Axial and (F) coronal contrast-enhanced postoperative MRI for patient two. Some residual tumor could not be safely resected at surgery because it was adherent to branches of the middle cerebral artery. (G) Coronal contrast-enhanced MRI and (H) noncontrast coronal CT for patient two, 5 years following incomplete resection, shows recurrence of the meningioma, with even more dense calcification. Histopathology following repeat surgery showed it had transformed to a World Health Organization grade II tumor.

A 7-year-old boy presented with a 9-month history of partial complex seizures. He was noted by his schoolteachers to have staring spells for a few seconds at a time, during which he was verbally unresponsive. He was thought to have attention deficit disorder or a behavioral abnormality, but EEG was performed after he described the sensation of olfactory auras at onset of these spells, and it confirmed seizures. An MRI showed a 4 × 5 × 4-cm mass in the left sylvian fissure region, very similar in appearance to the first patient ( Figs. 2A , 2B , and 2C ). It was more heavily calcified than the first patient's tumor on CT ( Fig. 2D ). He was referred for surgical evaluation and was noted to have a very mild right hemiparesis. He then underwent left frontotemporal craniotomy. The tumor was noted to be without any dural attachment and was found to be primarily intraparenchymal in location. A small incision was made in the anterior left temporal cortex, and the tumor was found 2 mm deep to the surface. The tumor was removed completely from the temporal lobe, but it also crossed into the frontal operculum, wrapping around the sylvian fissure and the major blood vessels within it. Surgery was stopped because of progressive cerebral edema before complete resection was achieved. A second surgery was performed at 2 weeks after the first through the same craniotomy, this time through a cortical incision in the anterior aspect of the frontal operculum, but the tumor was densely adherent to the MCA branches and a portion was left attached there to avoid injury to these vessels. Histologic examination showed fibrous meningioma with no mitotic figures, consistent with a WHO grade I tumor, and postoperative imaging showed only a small amount of residual tumor ( Figs. 2E and 2F ). The patient recovered well after surgery, including complete resolution of his right hemiparesis, without other neurologic deficits. His family moved out of the area and he was lost to follow-up for several years, but later returned for evaluation when his seizures recurred at about 5 years after surgery. Repeat MRI ( Fig. 2G ) showed a large tumor recurrence, measuring once again over 5 cm, this time with dense calcification of the entire tumor seen on CT ( Fig. 2H ).

Repeat craniotomy was performed, but the tumor was so densely calcified that it could only be removed by drilling it with a diamond-coated burr, a slow and tedious process, and only about half of the tumor could be safely removed without undue risk of injury to the MCA vessels. Postoperatively, he had a significant expressive aphasia that lasted almost 3 weeks before it resolved completely. Pathology from this recurrent tumor showed microscopic invasion of the pial surface, consistent with transformation to WHO grade II meningioma, but no increase in mitotic rate or nuclear atypia was seen. Further surgery was deemed very high risk for permanent speech and language deficits, and he underwent fractionated external beam radiation therapy instead. His tumor remains stable in size 2 years later, but he continues to have occasional seizures, despite medication.

### Patient Three

**Fig. 3:**
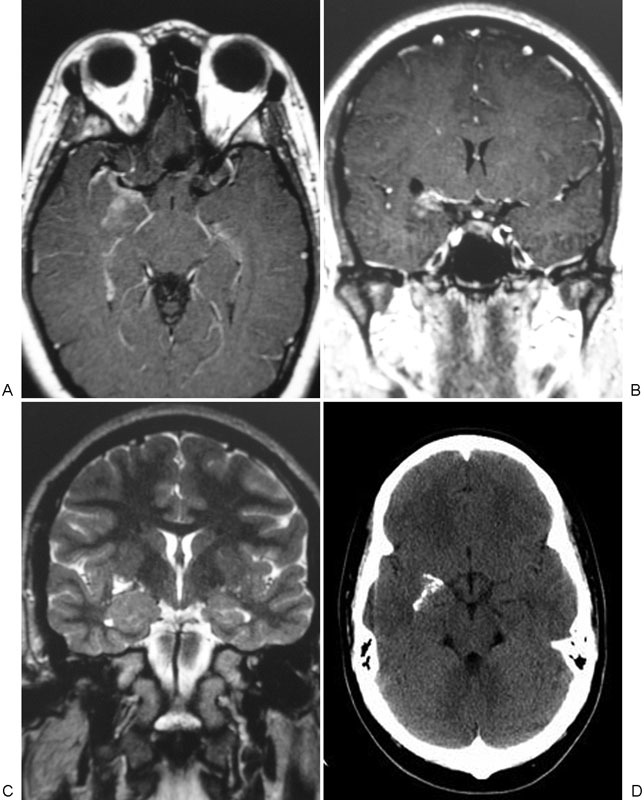
(A) Axial contrast-enhanced, (B) coronal contrast-enhanced, (C) coronal T2-weighted, and (D) axial noncontrast computed tomography preoperative images from patient three showing a partially calcified and minimally enhancing right medial temporal lobe/perisylvian mass. This result was concerning for a possible meningioma, but histopathology of the surgical specimen following right frontotemporal craniotomy and partial resection showed meningioangiomatosis.

A 16-year-old girl was referred for evaluation because of a history of complex partial seizures preceded by olfactory auras, which had initially occurred infrequently but then increased to daily episodes. After more than 6 months of symptoms, these were finally recognized as seizures and confirmed with EEG. MRI showed a small enhancing right temporal lobe intraparenchymal mass ( Figs. 3A , 3B , and 3C ), with some calcification noted on CT ( Fig. 3D ). Upon surgical consultation, her neurologic examination was intact, with normal cognition, and there were no findings suggestive of neurofibromatosis or any family history of it. Right frontotemporal craniotomy was performed, and trans-sylvian exposure of the medial temporal lobe revealed an abnormal appearance to the pial surface. A limited resection was performed of the mass, and histopathologic analysis showed findings consistent with MA. The patient recovered well postoperatively, with no neurologic deficits. Anterior medial temporal lobectomy and resection was discussed with her parents as a possible treatment option, but serial imaging was recommended instead. She has experienced excellent medical control of seizures since the surgery, and follow-up MRI at 5 years showed no further growth.

## Discussion

In this article, we describe two additional cases of meningiomas in children in the sylvian fissure region without dural attachment and one case of MA in the perisylvian region. All three presented with partial complex seizures that were misdiagnosed initially as behavioral disorders, which is not uncommon in children with such seizure types and can delay diagnosis and treatment. The rarity of meningiomas in this location and the lack of dural attachment can mislead surgeons to exclude this diagnosis prior to surgery, which may lead to incomplete surgical planning. In particular, the intraparenchymal location of the tumor in our second case caused great concern initially when the sylvian fissure was opened and no tumor was seen. In our review of the literature, this intraparenchymal location has been described in detail previously for five cases, 18 and with our additional case as the sixth, it constitutes nearly one quarter of all reported sylvian fissure meningioma cases. The intraparenchymal location complicated our second patient's surgery—a cortical incision in the temporal lobe was necessary to find and remove the tumor, and we stopped the surgery because of cerebral edema related to the additional parenchymal dissection and retraction. The patient was brought back for a second-stage surgery a few weeks later with good clinical outcome, and successful multistage surgery has been reported in other cases previously. 22 These tumors are often large at initial presentation, which can also make resection more challenging.

Microsurgical resection of these tumors has been reasonably successful in reducing seizures, especially when complete resection is achieved. 5 11 17 23 Our patient who underwent total resection of the meningioma has remained seizure-free for 10 years, but the other patient with subtotal resection has continued to experience seizures associated with tumor recurrence and progression from WHO grade I to grade II histology. Although complete resection was difficult in this second patient because of the intraparenchymal location, the main reason it was deemed high risk was dense adherence of the tumor to the distal MCA branches in the dominant hemisphere. This finding has been evident in many of the other cases described in the literature, 18 20 22 23 and it is the greatest challenge to safe removal of these tumors. The tumor was also densely calcified at recurrence, further increasing the risk of safe resection, which is not uncommon among the other reported cases as well. Adjuvant radiation therapy was used for this child, which will hopefully prevent or at least slow tumor growth in the future, but complete resection at initial surgery remains the goal. Postoperative neuropsychiatric testing in all of our patients demonstrated no significant cognitive deficits when compared with preoperative testing, indicating that surgery can be safely performed without undue risk to cognition, but hemiparesis and potentially aphasia remain the greatest risks.

Some authors postulate that these tumors arise from arachnoid cap cells within the Virchow-Robin space, 14 an extension of the subarachnoid space along the small arteries that penetrate into the brain parenchyma, which could explain the intraparenchymal location in a significant number of them, but the origin is not well understood. The histopathology of these tumors is somewhat varied, including transitional, psammomatous, meningothelial, and fibrous variants, and even a rhabdoid variant. Most are WHO grade I tumors, but a few atypical (WHO grade II) tumors and even one malignant (WHO grade III) tumor have been found. In our cases, one was a grade I tumor that was a mixture of fibrous and meningothelial cells, and the second a grade I fibrous tumor that recurred and became grade II when it showed invasion of the parenchyma.

MA can also occur in the medial temporal lobe/perisylvian location, as we describe in our third case. Most are sporadic, but some have been observed in patients with neurofibromatosis. 24 It has been classified histopathologically as a distinct entity from meningioma and has been noted to have a much more indolent clinical course and decreased tendency for growth. 25 26 27 These lesions are often also calcified, but some authors have tried to determine other imaging findings that distinguish them from meningiomas or other tumors, including with MRI, magnetic resonance spectroscopy, and positron emission tomography. 28 29 MA has a more gyriform hyperintensity on MRI fluid-attenuated inversion recovery sequences and tends to be smaller and have more heterogeneous contrast enhancement than meningiomas. 30 However, imaging alone cannot confirm the diagnosis, and biopsy is still necessary. Most cases have been followed with serial imaging, without significant clinical or tumor progression. Others have undergone surgical resection, though, with some improvement in seizures. 25 31 Interestingly, more cases have been reported in recent years of MA associated with meningiomas in the same patients. 28 32 33 34 Genetic studies have suggested that there may be an increased proliferative index in MA cells when they are part of a meningioma, and there appears to be a distinct phenotype in those that are associated with meningiomas compared with those that are not. 28 However, it is unclear if these cells arise de novo and then give rise to meningiomas or arise secondarily from the neoplastic cells of the meningioma. Expert histopathologic review is imperative to distinguish MA from intraparenchymal meningioma, and further genetic studies may allow a better understanding of their relationship.

## Conclusions

Meningeal tumors in the sylvian fissure region without dural attachments are extremely rare, but should be considered preoperatively in patients with an enhancing mass in this region, especially in a pediatric patient with complex partial seizures. The diagnosis may be delayed as the patients may be misdiagnosed with behavioral or cognitive disorders before the seizures are discovered, as in the patients in our series. EEG studies may confirm the seizure disorder, and MRI provides the diagnosis and important details regarding location, size, presence of edema, and so on. Preoperative diagnosis of meningioma remains difficult based on imaging alone, however. Craniotomy and resection remain the treatment of choice for these meningiomas and can be performed with minimal cognitive deficits and improvement of seizures in many cases. They may pose great surgical risk, though, when adherent to the MCA vessels or when heavily calcified, which increases the incidence of subtotal resection. Subtotal resection poses a higher risk of recurrence, and these patients should be followed with long-term serial imaging. MA appears to have a more benign clinical course and does not necessarily require complete resection but may be associated with a higher risk of meningioma formation.
